# Reexpansion pulmonary edema after treatment of simultaneous bilateral spontaneous tension pneumothorax

**DOI:** 10.1186/1749-8090-8-62

**Published:** 2013-04-04

**Authors:** Jong Bum Kwon, Si Young Choi, Chi Kyung Kim, Chan Beom Park

**Affiliations:** 1 Department of Thoracic and Cardiovascular Surgery, Daejeon St. Mary’s Hospital, The Catholic University of Korea, Daejeon, Republic of Korea; 2 Department of Thoracic and Cardiovascular Surgery, Uijeongbu St. Mary’s Hospital, The Catholic University of Korea, Uijeongbu, Republic of Korea; 3 Department of Thoracic and Cardiovascular Surgery, St. Paul’s Hospital, The Catholic University of Korea, Seoul, Republic of Korea

**Keywords:** Pneumothorax, Pulmonary edema, Chest tubes

## Abstract

We report a case of 46-year-old male with simultaneous bilateral spontaneous tension pneumothorax. Severe reexpansion pulmonary edema developed after bilateral tube thoracoscomy, but he was recovered after 2 days ventilator care. After bilateral wedge resection and talc pleurodesis, he was discharged without complications and had remained well and without recurrence during the 8-year follow-up.

## Background

Unilateral spontaneous pneumothorax is a common clinical problems, but simultaneous bilateral spontaneous pneumothorax is rare and can be fatal if it progress to tension pneumothorax [1]. Life-threatening presentation of simultaneous bilateral spontaneous tension pneumothorax is extremely rare. Early recognition of the condition and rapid decompression is necessary.

Herein, we present a case of reexpansion pulmonary edema followed by simultaneous bilateral spontaneous pneumothorax.

## Case presentation

**Figure 1:**
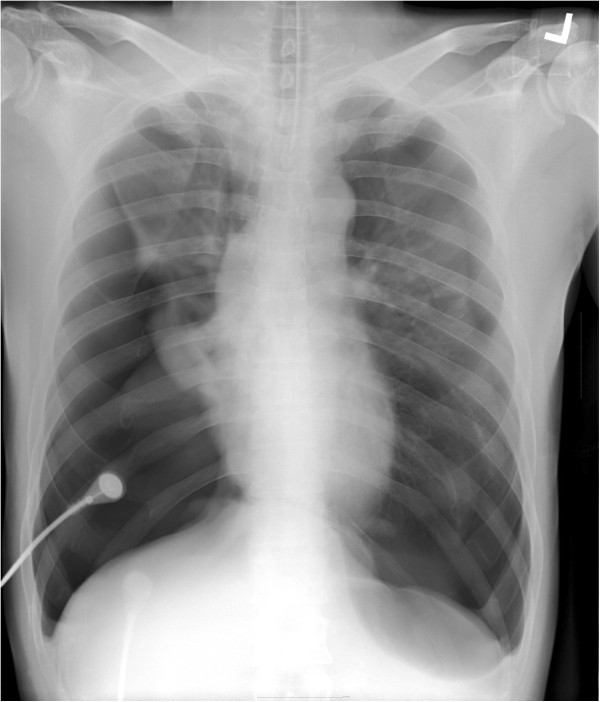
Chest x-ray showing bilateral pneumothorax with a flattened diaphragm and sharpened costophrenic angle.

**Figure 2:**
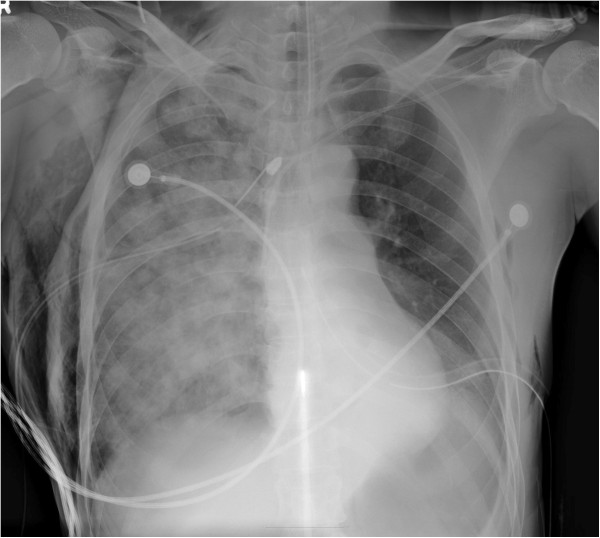
Severe reexpansion pulmonary edema on the right side of the chest was noted after tube thoracostomy.

A 46-year-old male with no history of underlying lung disease presented with severe shortness of breath. On arrival the patient was awake, his initial blood pressure was 240/150 mmHg, and respiratory rate was 38 breaths/min. Despite oxygen supplementation, his oxygen saturation dropped to 80% and he became cyanotic, suffered a seizure, and had no detectable pulse. Endotracheal intubation was performed immediately, followed by a chest radiograph, which showed bilateral tension pneumothorax (Figure 1). Emergent insertion of bilateral chest tubes stabilized the patient’s hemodynamic parameters. A subsequent chest radiograph revealed that both lungs were fully expanded; however, severe reexpansion pulmonary edema developed on the right side of the chest (Figure 2). The patient was transferred to the intensive care unit for ventilator care. On resolution of the reexpansion pulmonary edema, the patient was weaned from the ventilator and extubated on the second day of hospitalization. After removal of the tube thoracostomy in left side of his chest on the fourth day of hospitalization, the left lung was totally collapsed again, subcutaneous emphysema developed, and a chest tube had to be reinserted. Brain computed tomography (CT) for the evaluation of possibility of hypoxic brain damage, demonstrated no evidence of abnormalities, and high-resolution CT revealed multiple small air cysts and bullae in the bilateral upper lung fields. Single-stage bilateral wedge resection was performed on the 16th day of hospitalization, with talc pleurodesis. The postoperative course was uneventful and he was discharged without neurological sequelae. During 8 years after the operation, the patient had not experienced recurrent pneumothorax.

## Discussion

Simultaneous bilateral pneumothorax is usually caused by trauma, tumor, or iatrogenic causes, and the incidence of simultaneous bilateral spontaneous pneumothorax has been reported as 1.3%–4.0% [2,3]. Tension pneumothorax is an uncommon but life-threatening event that can result in significant cardiorespiratory collapse unless it is treated on an emergency basis. Although tension pneumothorax is not always accompanied by hemodynamic compromise, simultaneous bilateral tension pneumothorax, if untreated, progresses to rapid deterioration without exception.

Simultaneous bilateral tension pneumothorax has rarely been reported [1,4,5]. Such cases usually present in a critically ill and life-threatening state. Although immediate chest x-ray could confirm the existence of tension pneumothorax, the deteriorated condition of patients with simultaneous bilateral tension pneumothorax requires emergent endotracheal intubation and positive airway pressure ventilation aggravates the tension pneumothorax. Therefore, clinical suspicion of tension pneumothorax and immediate radiologic confirmation and emergent decompression could rescue these patients.

Mediastinal shifting with tracheal deviation is a typical finding of tension pneumothorax on chest x-ray, but this is not detected in cases of simultaneous bilateral tension pneumothorax, in which the diaphragmatic depression and sharpening of the costophrenic angle become more prominent. Reexpansion pulmonary edema is known to be a potentially lethal complication of lung reexpansion after tube thoracostomy. Factors that have been postulated to contribute to the development of reexpansion pulmonary edema were increased pulmonary vascular permeability, chronicity of pulmonary collapse, application of negative suction pressure, decreased surfactant activity, and pulmonary artery pressure change [6,7]. As in our case, rapid decompression of tension pneumothorax gives rise to the development of reexpansion pulmonary edema. However, this can be recovered successfully if it combined with other comorbidities. It is interesting that reexpansion pulmonary edema was developed on the right side after the bilateral chest tube insertion. We speculate that the larger size of pneumothorax on the right side might be associated with the unilateral development of reexpansion pneumothorax. Although emergent needle decompression could be considered in the management of tension pneumothroax, it failes to restore cardiac output [1]. The failure of needle thoracentesis to relieve tension is related to the length of cannula, the chest wall thickness, or the insertion technique [8-10]. Therefore, chest tube drainage should be performed even after needle thoracentesis, and definitive surgical resection of any lung bullae and chemical pleurodesis should be considered for the prevention of recurrent tension pneumothorax.

## Conclusion

The present case indicates that while simultaneous bilateral spontaneous tension pneumothorax is extremely rare, it induces life-threatening conditions in patients. Early recognition and immediate treatment is mandatory for saving the patient’s life. Although reexpansion pulmonary edema develops after tube thoracostomy in bilateral tension pneumothorax, it does not exacerbate the clinical course.

## Consent

Written informed consent was obtained from the patient for publication of this Case report and any accompanying images. A copy of the written consent is available for review by the Editor-in-Chief of this journal.

## Abbreviations

CT: Computed tomography

## Competing interests

The authors declare that they have no competing interests.

## Authors’ contributions

JBK contributed to conception and design of the manuscript. SYC contributed to writing the manuscript. CKK involved in the critical revision of the article. CBP was responsible for the integrity of the work and edited manuscript. All authors read and approved the final manuscript.
